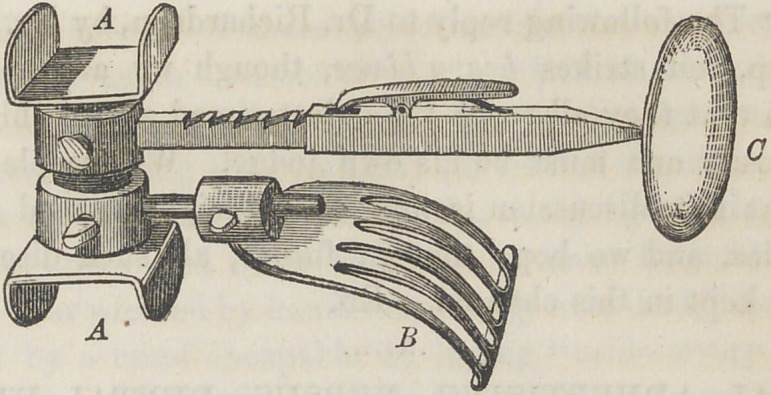# Dentist’s Assistant

**Published:** 1859-07

**Authors:** J. Taft


### DENTIST'S ASSISTANT.

BY J. TAFT.

THE accompanying cut represents a very ingenious and useful instrument, invented by Dr. C. C. THOMAS, of Natchez, Miss. The instrument is to be placed in the mouth of the patient, while the teeth are being filled, particularly the inferior molars. The object of the instrument is threefold: to keep the jaws asunder, the tongue down, and the cheek distended. The instrument is so made and articulated, that the distension of the jaws can be graduated according to the size of the mouth. The jaws rest upon it at *A*, so that they do not become fatigued by the mouth being kept open for a long time. The tongue compressor *B*, can be adjusted to any desired position, either backward, forward, or extended. The cheek-distender, *C*, can be extended so as to press the cheek entirely out of the way; it can also be rotated on the main shaft, and the part in contact with the cheek thrown back or forward, as the case may require. The whole is made of silver, and is very neat. When properly adjusted in the mouth, the operator has free use of both hands in manipulations.

It is well to pack round and on the tongue, folds of paper or napkin, then put the tongue-holder upon these; and in a great many cases, there will be no discharge of saliva from the sublingual or submaxillary ducts, even during a protracted operation; and in every case, it will be very much checked. It is a very valuable instrument, and one that I would not be without for thrice the cost. TOLAND furnishes them, at $10 each.

☞ The following reply to Dr. Richardson, by Dr. DILLS, is sharp, and strikes *heavy blows*, though we are not quite certain that they all reach the object aimed at; of this, however, every one must be his own judge. We are pleased to see that this discussion is *now* on principle instead of personalities, and we hope that in future, all such discussions will be kept in this channel.—ED.

## Figures and Tables

**Figure f1:**